# Relevant factors of posterior mandible lingual plate perforation during immediate implant placement: a virtual implant placement study using CBCT

**DOI:** 10.1186/s12903-022-02696-z

**Published:** 2023-02-06

**Authors:** Yingjia Sun, Sai Hu, Zhijian Xie, Yiqun Zhou

**Affiliations:** 1 Stomatology Hospital, School of Stomatology, Zhejiang University School of Medicine, Zhejiang Provincial Clinical Research Center for Oral Diseases, Key Laboratory of Oral Biomedical Research of Zhejiang Province, Cancer Center of Zhejiang University, Hangzhou, 310000 People’s Republic of China; 2 The Fourth Affiliated Hospital, Zhejiang University School of Medicine, N1 Shangcheng Road, Yiwu, 322000 Zhejiang People’s Republic of China

**Keywords:** Cone-beam computed tomography, Immediate implant placement, Mandible, Molar, Complication

## Abstract

**Background:**

To explore the influence of cross-sectional type and morphological parameters at the mandibular molar sites on lingual plate perforation (LPP) during the immediate implant placement (IIP).

**Methods:**

181 implants were virtually placed in the mandibular molar sites on the cone beam computed tomography (CBCT). Each cross-section of the implantation site was divided into the Undercut (U)/Parallel (P)/Convex (C) types. Morphologically relevant parameters were measured on the cross-sections, including width of the upper end (Wb), width of the lower end (Wc), vertical height (V), angle between the natural crown axis and the alveolar bone axis (∠β), LC depth (LCD), LC height, and angle between the horizontal line and the line connecting the most prominent point and the most concave point of lingual plate (∠α). Besides, the distance from the end of the virtual implant and the lingual bone plate of the cross-section (D_IL_) was calculated. Relationships between all the morphologically relevant parameters and the D_IL_ were further analyzed.

**Results:**

A total of 77 (42.5%) cross-sections were classified as U-type, which was the most common one, accounting for 63% of the second molar regions. All LPP cases and most of the nearly LPP (87.9%) cases occurred at the U-type cross-sections, and the relationship between the D_IL_ and the morphological parameters can be expressed by a multivariate linear equation.

**Conclusions:**

The occurrence rate of U-type cross-sections in the second molar region was very high, and the risk of LPP should be considered during IIP. Except for the U-type, significant large LCD, small Wc, and large ∠β were the important relevant factors. CBCT and multivariate linear equations could help to assess the LPP risk and provide a reference for implant placement design pre-surgery.

## Background

Extraction of a first or second molar due to dental caries or periodontitis is a common cause of clinical dentition defects [1]. Dental implants have become the optimal treatment for tooth replacement [2]. Immediate implant placement (IIP) can better satisfy the urgent recovery of posterior occlusal function [3–5]. However, inserting implants into the mandibular lingual cortex, or even penetrating the lingual plate is one of the serious complications that may result in hemorrhages, nerve damage, inflammation, and infection [6, 7]. It is reported statistically that the incidence of lingual plate perforation (LPP) can reach 1–2%, and the real risk is thought to be even higher [8–10].

Lingual concavity (LC) is a depression on the medial surface of the mandible [11] and is a highly vascularized area containing important nerves and arteries [12]. Deep LC is found to be the main relevant factor of LPP during implant placement [13]. Additionally, the cross-sectional shape of the posterior mandible has also been found to influence LPP occurrence [10, 14, 15]. Many studies [9, 16, 17] have classified the cross-section of the posterior mandible as Undercut (U), Parallel (P), or Convex (C)-type [8] and suggested that the U-type could increase the LPP risks during the IIP. However, the implant sites selected in these studies were all after tooth extraction and bone reconstruction [18], which affected jaw morphology. They could not reflect the influence of primitive jaw morphology on LPP.

Nowadays, implant design software with DICOM data from cone-beam computed tomography (CBCT) can simulate implant effects before surgery [19]. It is beneficial to select CBCT with natural molars to eliminate the influence of bone remodeling. A natural crown can also be used as “restoration” to determine the direction of implant placement [10, 20, 21]. In this study, patients with natural molars were selected as research objects, and simulated implants were placed on their CBCT to observe the occurrence of LPP. Except for the measurements about LC and cross-sectional shape, the angle between the direction of implant placement and the long axis of the alveolar bone was also suggested to affect the probability of bone plate perforation in a study by Kong et al. [21] and our previous clinical study [22]. In this study, the direction of virtual implant placement is consistent with the natural crown axis. We defined the angle between the natural crown axis and the alveolar bone axis as ∠β and also included ∠β in the morphological measurements. Meanwhile, an equation was intended to be constructed to study the influence of the above multiple factors on LPP. This study aims to identify the key morphological features of mandibular cross-sections with high perforation risks in order to assist in the preoperative implant design, such as implant diameter, implant length, and implantation angle [23].

## Methods

### Patient inclusion criteria

Patients with CBCT data were included according to the following selection criteria:

- Age of patients ≥ 18 years;
- The intact first molar or second molar and its adjacent tooth on at least one side of the mandible;
- The curvature of the Spee Curve of the mandibular dentition within the normal range;
- No obvious dislocation of the molar area, no abnormal alveolar bone resorption in the investigated area, and no obvious pathology lesions in the apical and periodontal area;
- Obvious inferior alveolar canal (IAC) and the outline of the mandible on CBCT images; and
- No scattering or beam-hardening artifacts or other reasons on CBCT images.

The first molar or the second molar on any side of the mandible was selected upon it met the requirements. If both sides in the same patient satisfied the selection criteria, one side was selected randomly for inclusion.

Data of patients in the Stomatology Hospital, School of Stomatology, Zhejiang University School of Medicine, Hangzhou, China between June 2018 and June 2020 were collected. The ethical approval was provided by the Ethics Review Board of the Stomatology Hospital, School of Stomatology, Zhejiang University School of Medicine (No. ZHUSSIRB-2021-33R). Every patient signed an ethical board-approved written informed consent form.

### CBCT parameters

Head of the patient was adjusted to a uniform standard for CBCT examination, and Frankfort horizontal plane in the CBCT was corrected to be parallel to the real horizontal plane. All CBCT images were obtained with a field of view (FOV) of 11 × 8 cm^2^ by the NewTom 3G machines (QR, Verona, Italy) at the Department of Radiology, the Stomatology Hospital, School of Stomatology, Zhejiang University School of Medicine, Hangzhou, China. The imaging parameters were set at 18.66 mAs, 120 kVp, a resolution of 0.4 mm, and a scan time of 20 s. Images were reconstructed based on the three-dimensional (3D) CBCT data with an implant planning software program (Simplant, MATERIALISE Clinical Service Inc., Leuven, Belgium) and were measured.

### Virtual implant placement and data acquisition

#### Virtual implant placement

**Fig. 1:**
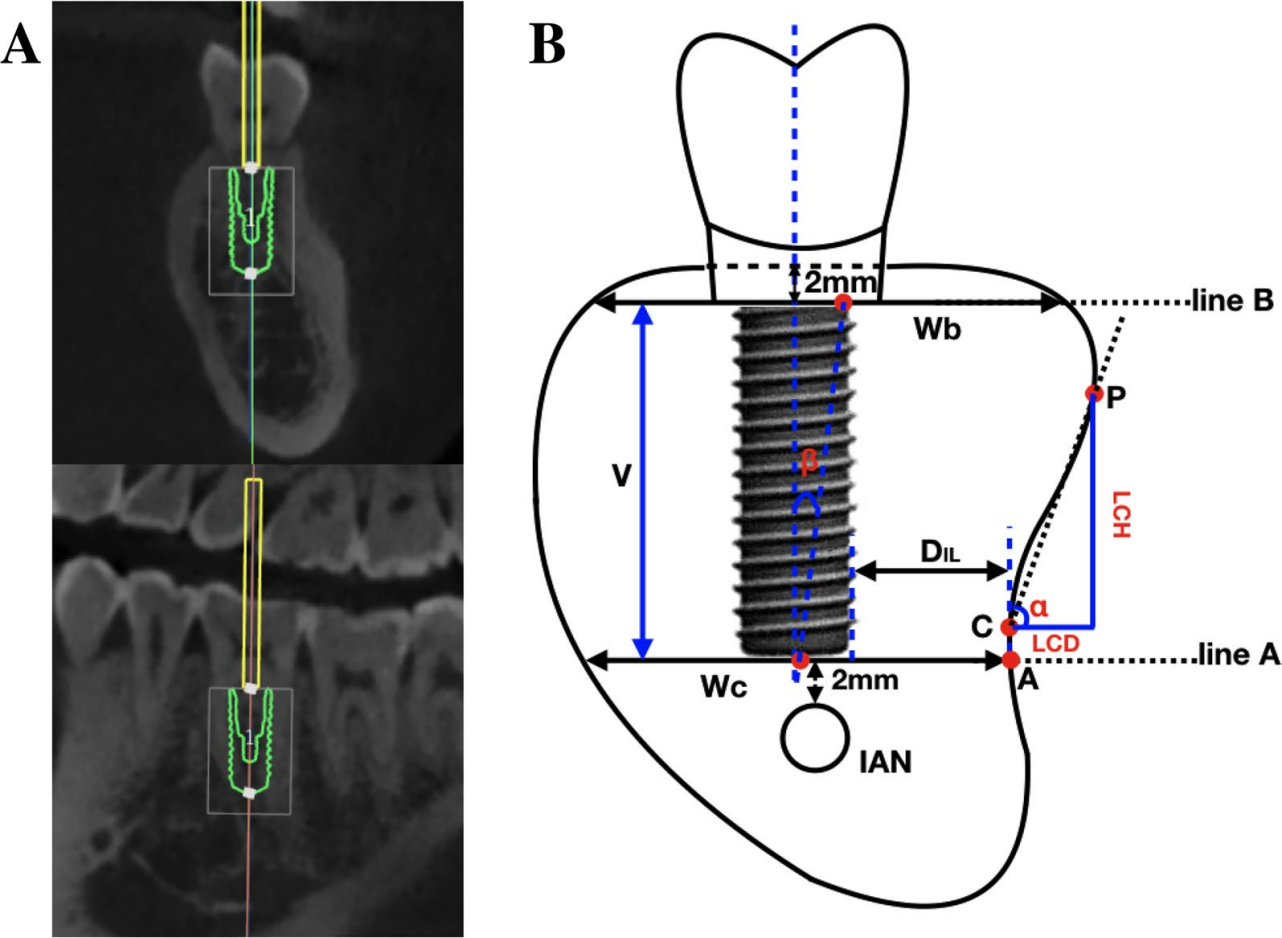
Virtual implant placement and data measurements: **a** Schematic diagram of virtual implant placement. The axis of the implant was along the long axis of the natural crown and was verified on 3D images. **b** Cross-sectional morphology-related measurements and implant-related parameters

In this study, two widely used diameter types (4.1 mm and 4.8 mm) of screw thread column form implants were selected [24]. The implants were virtually placed along the center vertical axis of the natural crown, and the orientation of the implants was verified in 3D [10]. The implant platform was further adjusted to a horizontal level of 2 mm apical to the crest level and the implant tip was at the horizontal level of 2 mm above the upper edge of the IAC (Fig. 1a).

### Cross-sectional morphology-related measurements

**Fig. 2:**
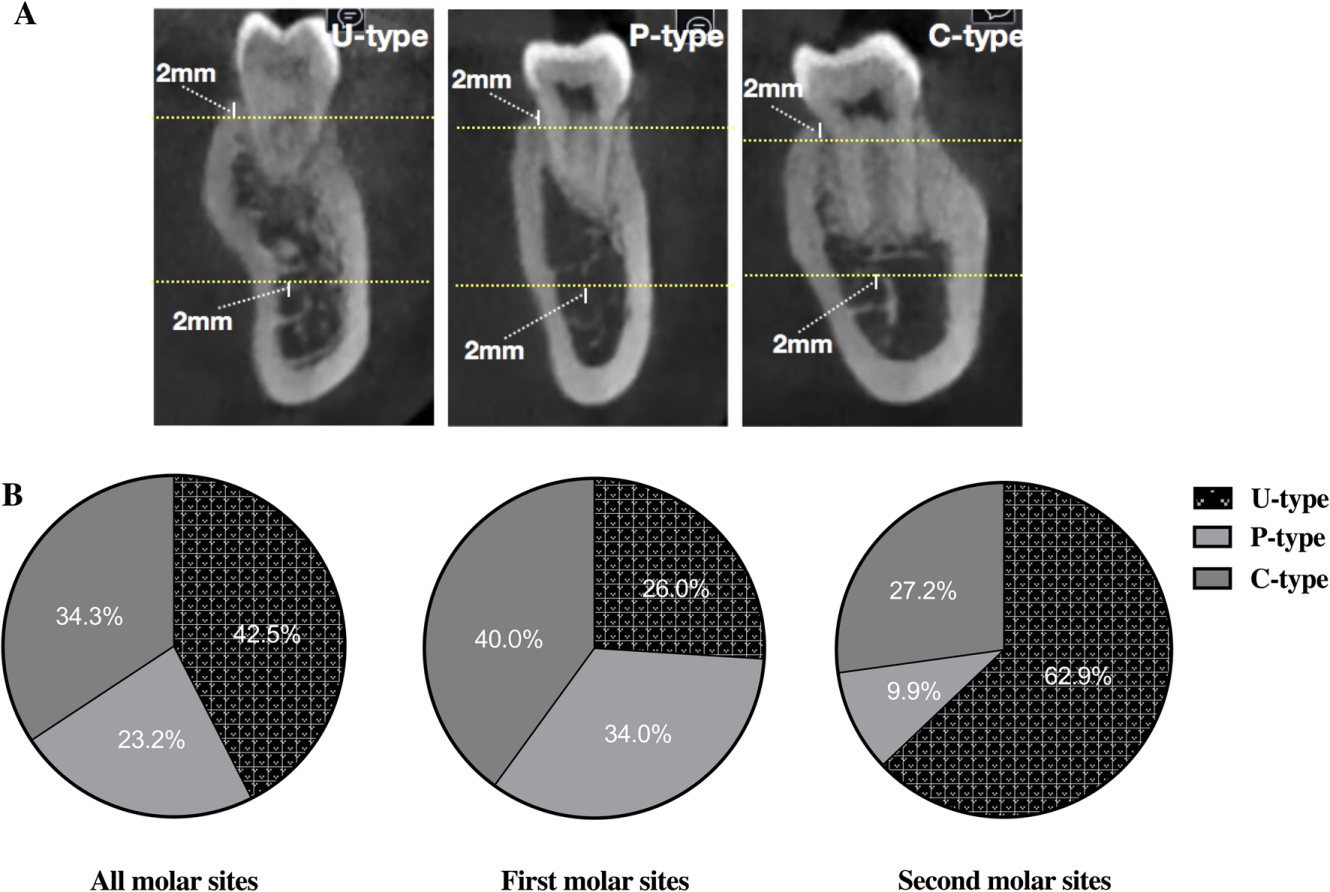
Three types of cross-sectional posterior mandibular morphology: **a** Schematic diagram of U, P, and C-type. **b** Distribution of the U-, P-, C-type in cross-sections of dentulous molar sites

The coronal cross-sections of the implantation sites were aligned with the long axis of the mandibular first or second molar crown and passed through the center point of the crown. Line A was a horizontal line 2 mm above the upper edge of the IAC. The intersection point of Line A and the lingual bone plate of the cross-section was marked as point A. The width of the mandible cross-section on the Line A level was defined as Wc. Line B was a horizontal line 2 mm apical to the crest, where the width of the mandible cross-section was set to Wb [8]. The vertical distance between Line A and Line B was represented by V, referring to the height of the ridge (Fig. 1a). The mandibular cross-sectional morphology from Line A level to Line B was categorized into one of the following three groups: convex (C-type): the lingual plate of the cross-section was round and the base of the ridge was wider than the crest, parallel (P-type): the lingual plate of the cross-section was parallel or nearly parallel to the buccal plate without no obvious lingual undercut, and undercut (U-type): a distinct undercut was found on the lingual plate in the cross-section and the base of the ridge was narrower than the crest [8] (Fig. 2a). ∠β (°) in the present study was defined as the angle between the long axis of the natural crown and that of the alveolar bone cross-section (connecting the line through the midpoints of Wb and Wc). If the long axis of the natural crown was on the lingual side of the long axis of the alveolar bone cross-section, ∠β (°) > 0, otherwise, ∠β (°) < 0. The most concave point in the lingual side of the U-type cross-section was point C, and its prominent point was denoted as point P. ∠α referred to the angle between the line connecting point P and point C and the horizontal line through point C. The horizontal and vertical distance between point P and point C was the LC depth (LCD) and LC height (LCH), respectively [8] (Fig. 1b).

### Implant-related measurements

Distance from surface of the lingual bone plate to the end edge of the virtual implant was defined as D_IL_ (mm). The distance was defined as D_IL_ (⌀ = 4.1 mm) if the implant was virtually placed with a diameter of 4.1 mm, while D_IL_ (⌀ = 4.8 mm) was applicable at a diameter of 4.8 mm. If the lingual bone plate was perforated by the implant, D_IL_ (mm) was recorded as < 0, otherwise, it was > 0 (Fig. 1b).

All simulated implantation and measurement procedures were performed in the implant design software (Simplant, MATERIALIZE Clinical Service Inc., Leuven, Belgium). CBCT files (in DICOM format) were imported into the software to be measured by two examiners (oral and maxillofacial radiologists) who were blinded to experimental design and had more than five years of clinical experience in CBCT evaluation. The cross-sections and various measurement parameters based on CBCT images were anatomically classified by using an intra-examiner calibration to assess the data reliability. After that, the images were evaluated individually by two inspectors, and any disagreements in the interpretation of the images were discussed until a consensus was reached [20].

### Statistical analysis

The data were statistically analyzed using Statistical Package for the Social Sciences (SPSS) (version 23.0; SPSS Inc., Chicago, IL, USA). Frequency was employed to exhibit the classification of the cross-sectional morphology of the mandible. The frequencies of three types among the different molar sites were compared by the chi-squared (X^2^) test. Continuous variables were presented as the mean ± standard deviations (SDs). The morphological comparisons between the first and the second molar site and between the perforated sites and non-perforated sites were performed by student’s t-test for statistical analysis. Multivariate linear regression was employed to analyze the linear relationships between morphological parameters with the distance of the implant tip relative to the lingual bone plate. *P < 0.05 meant the difference was statistically significant.

## Results

### Distribution of cross-sections with three morphological types

All selected CBCT data were obtained from 63 men and 40 women with a mean age of 33.4 ± 7.6 years old. A total of 181 cross-sections of the mandibular molar region were obtained from CBCT images, including 100 (55.2%) and 81(44.8%) in the first and second molar region, respectively. The inter-examiner reliability of each parameter on cross-sections was higher than 0.90 for two examiners. The U-type was the most common (42.5%) of the three different types of cross-sections. In the cross-sections of the second molar region, the proportion of U-type was the largest, reaching 63.0%. There were significant differences in the distribution of cross-sectional morphology between different molar sites (*P* < 0.05) (Fig. 2b).

### Comparison of morphological measurements and D_IL_ in different molar sites

**Fig. 3:**
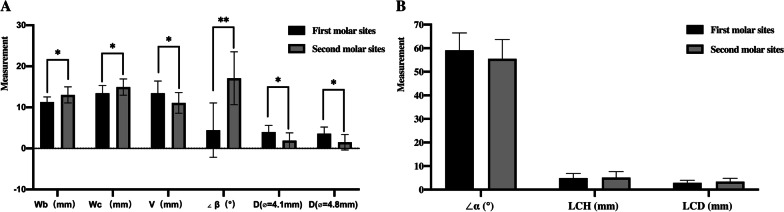
Cross-sectional morphology-related measurements and implant-related parameters: **a** Features of cross-sections and the distance between the lingual bone plate and the virtually placed implant in different molar sites. **b** Features of the LC in U-type cross-sections in different molar sites. * (black): significant difference in measurements between two tooth sites

Among all objects, the mean values of Wb, Wc, V, and ∠β were 12.07 ± 1.78 mm, 14.11 ± 2.06 mm, 12.39 ± 3.01 mm, and 10.09 ± 9.07°, respectively. Besides, the mean values of D_IL_ (⌀ = 4.1 mm) and D_IL_ (⌀ = 4.8 mm) were 3.06 ± 2.03 mm and 2.67 ± 2.02 mm, respectively. The differences were statistically significant (*P* < 0.05) in all the above measurements between the first and second molar sites and the ∠β turned a more significant difference (*P* < 0.01) (Fig. 3a). The mean values of LCD, LCH, and ∠α in the cross-sections of second molar sites were 3.37 ± 1.44 mm, 5.12 ± 2.52 mm, and 55.50 ± 8.15°, respectively. It showed a deeper fossa of the lingual plate in the second molar sites when compared to that in the first molar sites but not significantly (Fig. 3b).

### *Distribution of cross-sections with DIL* < *0 mm, DIL* ≤ *1 mm, or DIL* ≤ *2 mm*

Table 1 showed the number of cases with D_IL_ < 0 mm, D_IL _≤ 1 mm, or D_IL_ ≤ 2 mm in different tooth sites, with different implant diameters and cross-sectional morphological types. When ⌀ = 4.1 mm and ⌀ = 4.8 mm implants were virtually placed in all the molar sites, all perforation cases (D_IL_ < 0 mm) occurred in the U-type cross-sections. Among them, the ⌀ = 4.8 mm implants virtually placed in U-type cross-sections of the second molar sites showed the highest possibility of LPP (22.2%). A total of 8.0–12.0% and 46.9–53.1% of cross-sections in the first and second molar sites were found at D_IL_ ≤ 2 mm, respectively. D_IL_ ≤ 2 mm indicated that risk of the implant entering the cortical bone during the IIP is high, even causing LPP. Among the cross-sections with D_IL_ ≤ 2 mm, the proportion of U-type was the largest (83.3–100%). Meanwhile, a total of 30.8–38.5% and 70.6–76.5% of the U-type cross-sections in first and second molar sites were found with D_IL_ ≤ 2 mm, respectively (Table 1).

**Table 1:** The number of cases with D_IL_ < 0 mm, D_IL_ ≤ 1 mm, or D_IL_ ≤ 2 mm in different molar sites

Implant size	Distance threshold	First molar					Second molar				
		C-type	P-type	U-type	Total	Percent A (%)	C-type	P-type	U-type	Total	Percent A (%)
		40	34	26	100		22	8	51	81	
	≤ 2 mm	0	0	8	8	100.0	0	2	36	38	94.7
⌀ = 4.1 mm	≤ 1 mm	0	0	5	5	100.0	0	0	21	21	100.0
	< 0 mm	0	0	2	2	100.0	0	0	13	13	100.0
Percent B (%)		0.0	0.0	30.8	8.0		0.0	25.0	70.6	46.9	
		40	34	26	100		22	8	51	81	
	≤ 2 mm	1	1	10	12	83.3	2	2	39	43	90.7
⌀ = 4.8 mm	≤ 1 mm	0	0	7	7	100.0	0	0	31	31	100.0
	< 0 mm	0	0	2	2	100.0	0	0	18	18	100.0
Percent B (%)		0.0	0.0	38.5	12.0		9.1	25.0	76.5	53.1	

Percent A, the number of cases with D_IL_ < corresponding threshold distance in U-type subjects/the number of cases with D_IL_ < corresponding threshold distance in total subjects

Percent B, the number of cases with D_IL_ ≤ 2 mm in C/P/U-type subjects/the number of all the C/P/U-type subjects

### Comparison of morphological measurements between perforated and non-perforated sites

There were statistically significant differences in the Wb, Wc, and ∠β between the perforated sites and non-perforation sites (*P* < 0.01) (Table 2). Perforated sites were significantly wider at Line B level of the cross-sections but narrower at Line A level compared to the non-perforated sites. Perforated sites showed a significantly larger ∠β compared to the non-perforated sites. For all the U-type cross-sections, perforated sites exhibited a higher LCH and deeper LCD significantly (*P* < 0.01) (Table 2).

**Table 2:** Comparison of morphological parameters between perforated sites and non-perforated sites

	⌀ = 4.1 mm implants				⌀ = 4.8 mm implants			
Measurement	Perforated	Non-perforated	Total	*P*	Perforated	Non-perforated	Total	*P*
Wb (mm)	13.70 ± 1.94	11.93 ± 1.70	12.07 ± 1.78	< 0.001	13.50 ± 2.00	11.90 ± 1.68	12.07 ± 1.78	< 0.001
Wc (mm)	12.68 ± 1.93	14.24 ± 2.03	14.11 ± 2.06	0.005	12.95 ± 1.88	14.26 ± 2.04	14.11 ± 2.06	0.007
V (mm)	11.89 ± 2.21	12.43 ± 3.07	12.39 ± 3.01	0.501	11.77 ± 2.18	12.47 ± 3.10	12.39 ± 3.01	0.331
∠ β (°)	18.42 ± 5.05	9.34 ± 8.98	10.09 ± 9.07	< 0.001	18.61 ± 4.98	9.04 ± 8.91	10.09 ± 9.07	< 0.001
LCH (mm)	7.26 ± 2.90	4.53 ± 1.80	5.06 ± 2.31	< 0.001	6.66 ± 2.82	4.50 ± 1.82	5.06 ± 2.31	< 0.001
LCD (mm)	4.84 ± 1.35	2.81 ± 1.00	3.21 ± 1.34	< 0.001	4.60 ± 1.32	2.72 ± 0.95	3.21 ± 1.34	< 0.001
∠α (°)	55.04 ± 6.73	57.12 ± 8.30	56.72 ± 8.02	0.370	53.89 ± 6.76	57.71 ± 8.25	56.72 ± 8.02	0.067

### The linear relationship between D_IL_ and all the related morphological parameters

**Fig. 4:**
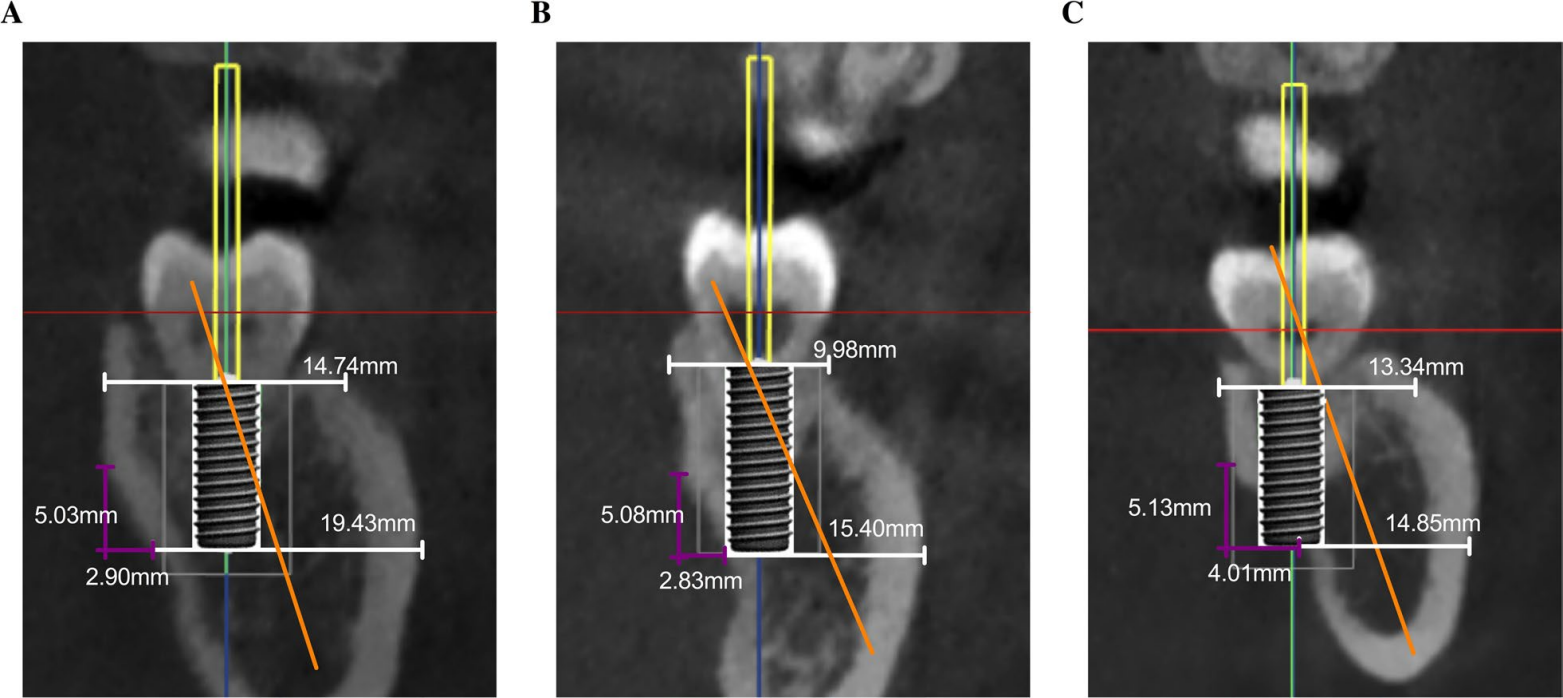
Comparison of features between the perforated sites and non-perforated sites: **a** Typical schematic image of non-perforated cross-sections. **b** A typical schematic image of non-perforated cross-sections but the implant into the lingual bone cortex. **c** A typical schematic image of perforated cross-sections

Linear relationships and two linear regression equations were found between D_IL_ (⌀ = 4.1 mm) or D_IL_ (⌀ = 4.8 mm) and all the related parameters in the U-type cross-sections (Table 3). The coefficients of LCD were much greater than those of Wc and ∠β regardless of implant diameter (Table 3). Three typical schematic images of “non-perforated sites”, “non-perforated sites but the implant into the cortex”, and “perforated sites” reflected the influence of the varying LCD, Wc, and ∠β on the LPP. The typical schematic image of perforated cross-sections showed greater LCD and ∠β and a smaller Wc compared to the non-perforated cross-sections (Fig. 4).

**Table 3:** Analysis of the linear relationship between all the related morphological parameters with D_IL_(coefficient*)

Model	Nonstandardized coefficient		Standardized coefficient		
	B	Standard error	Beta	t	*P*
1					
(Constant)	1.434	3.417		0.420	0.676
Wb (mm)	− 0.045	0.100	− 0.043	− 0.457	0.649
Wc (mm)	0.204	0.096	0.210	2.139	0.036
V (mm)	0.073	0.062	0.113	1.167	0.247
∠ β (°)	− 0.063	0.025	− 0.247	− 2.474	0.016
LCH (mm)	0.099	0.272	0.119	0.363	0.718
LCD (mm)	− 0.998	0.437	− 0.698	− 2.284	0.025
∠α(°)	0.011	0.052	0.045	0.209	0.835
2					
(Constant)	0.959	3.460		0.227	0.782
Wb (mm)	− 0.043	0.101	− 0.040	− 0.425	0.672
Wc (mm)	0.204	0.097	0.209	2.113	0.038
V (mm)	0.072	0.063	0.111	1.137	0.259
∠ β (°)	− 0.061	0.026	− 0.247	− 2.370	0.021
LCH (mm)	0.086	0.275	0.103	0.311	0.757
LCD (mm)	− 0.985	0.442	− 0.686	− 2.226	0.029
∠α(°)	0.012	0.052	0.051	0.234	0.816

Model 1 (U-type, R^2^ = 0.612): D_IL_ (⌀ = 4.1 mm) =   − 0.998LCD + 0.204Wc − 0.063∠ β

Model 2 (U-type, R^2^ = 0.612): D_IL_ (⌀ = 4.8 mm) =   − 0.985LCD + 0.204Wc − 0.061∠ β

## Discussion

IIP in the posterior mandible can provide faster recovery of masticatory function in selected scenarios [3, 25, 26]. However, surgeons need to try their best to prevent serious complications such as LPP. In the present study, the dentulous mandible was selected and simulated implants were placed under the guidance of natural crowns. This study comprehensively analyzed the primitive morphology of posterior mandible and its effect on the occurrence of LPP during immediate implant surgery. The results showed that U-type cross-sections accounted for the largest proportion at 42.5% in the dentulous posterior mandible, and LPP all occurred in the U-type cross-sections after virtual implant placement. LPP and nearly LPP were most likely to occur at second molar sites with U-type cross-sections. Of the morphological measurements, large LCD, ∠β, and small Wc presented significant negative impacts on LPP.

Tooth extraction or tooth loss may affect the distribution of each cross-sectional type by bone remodeling. It was reported in a study by Chan et al. [8] that 66% of edentulous molar sites have U-type cross-sections, as well as in a study by Aparicio et al. [17] that 64.2% do. However, the proportion of U-type cross-sections in dentulous molar sites was lower, only 46.7% in the previous study [20] and 42.5% in this study, indicating a lower risk of LPP during immediate implant surgery.

Tooth sites affect the distribution of each cross-sectional type due to different morphological characteristics. Cross-sections in the second molar sites were mostly U-type (62.9%) compared to those in the first molar sites (26.0%) (Fig. 2), which is consistent with findings in prior studies [10, 16, 27]. Additionally, cross-sections in the second molar sites showed higher implant perforation risk with a significantly smaller value of D_IL_ (⌀ = 4.1 mm) or D_IL_ (⌀ = 4.8 mm) compared to those in the first molar sites. Such consequences might result from different initial bone profiles between two molar sites. Cross-sections in the second molar sites showed significantly larger Wb, Wc, and ∠β while a significantly smaller V compared to those in the first molar sites. If the cross-sectional shape could be roughly represented as a quadrilateral, it in the second molar sites was like a squat and oblique quadrilateral and more likely to show the LC, while that in the first molar sites was more like a tall, thin, and straight quadrilateral. More attention should be paid to immediate implants placed in the second molar sites and other tooth sites with similar morphological features.

Virtual implants exhibited all LPP in U-type cross-sections, and nearly all almost-perforation cross-sections with DIL ≤ 1 mm or DIL ≤ 2 mm were also U-type. Kim et al. [28] measured the cortical bone thickness in the mandibular lingual plate using computed tomography and found it was more than 2 mm in all posterior cross-sections. If the instrument touches or even enters the cortical bone during the reaming, the dexterity and stability of the operator's hands will be greatly affected, and the excessive heat generated in the local area may damage the surrounding tissue. In addition, the actual implantation situation is more restricted than the preoperative simulation [29]. This computer simulation research required statistical analysis of not only the cross-sections with D_IL_ < 0 mm, but also those with D_IL_ ≤ 1 mm or D_IL_ ≤ 2 mm implying the potential risk of cortical bone invasion. Samples were grouped according to tooth sites and cross-sectional type, and U-type cross-sections in second molar sites had the smallest D_IL_. Clinically, the risk of lingual plate perforation at these sites could be reduced by choosing smaller diameter (⌀ = 4.1 mm) implants or root-shaped implants.

Compared to the non-perforated cases, the perforated cases most occurred in the cross-sections with greater Wb and ∠β but a smaller Wc and in the U-type cross-sections with larger LCH and LCD. The multiple linear regression equation further highlighted the parameters with strong correlations with D_IL_ (⌀ = 4.1 mm) or D_IL_ (⌀ = 4.8 mm). The results revealed that in the U-type cross-sections, the coefficient of LCD and Wc were much greater, followed by that of ∠β. The angle between the long axis of the natural crown and that of the alveolar bone was named ∠β in this study. For the first time, the line through the midpoints of Wb and Wc was defined as the long axis of the alveolar bone, which may be closer to clinical practice than previously reported [21]. To better reflect the concept of restoration-guided implant placement [21, 30], virtual implants were placed with their long axis aligned with natural crowns [16]. The results suggested that ∠β had a critical impact on LPP, which was of clinical significance. If LC is deep in posterior mandible, the long axis of implants can be modified to move the apex of implants buccally to avoid LPP.

Despite the relatively low probability of lingual perforation during IIP, its consequences can be serious and even life-threatening once it happens [31, 32]. Clinicians can use bone augmentation [33], shorten implant length, decrease the diameter of implants, and tilt implants [34] to lower the incidence of LLP in high-risk regions [35]. Furthermore, the tapered implants may be better applicable for the U-type ridge in the second molar site to avoid lingual perforation around the apical area. Although this virtual study was carried out on the dentulous mandible, the conclusions and suggestions were also helpful for conventional implant surgery.

This study still had several limitations. Firstly, it was a "virtual" study. Placing an immediate implant in a post-extraction molar site was, therefore, a complex and challenging procedure. Due to the different shapes of the tooth extraction socket, the implantation direction of clinical immediate implantation is not limited to the long axis of the natural crown. It is necessary to choose a suitable direction that can provide initial bone stability for the implant. Moreover, the inter-observer variations for measurements were high, so the minor discrepancies may occur in determining the position of the virtual implant. For these reasons, the findings of present study should be interpreted carefully and supported by well-designed and prospective controlled trials with long-term follow-up. In future research, a more systematical evaluation system could be continued to explore and expand the measurement parameters to obtain more reliable calculation equations for reference.

## Conclusion

The cross-sectional type, tooth sites, LCD, Wc, and ∠β were all associated with LPP during IIP. CBCT images and a multivariate linear equation could be used to assess the risk of LPP before surgery. As well, the diameter, length, and direction of implants can be adjusted properly to minimize unpleasant complications.

## Data Availability

The data used and analyzed during the current study are available from the corresponding author on reasonable request.

## Abbreviations

CBCT: Cone beam computed tomography
IIP: Immediate implant placement
IAC: Inferior alveolar canal
LC: Lingual concavity
LPP: Lingual plate perforation
